# Regulation and biological role of the peptide/histidine transporter SLC15A3 in Toll-like receptor-mediated inflammatory responses in macrophage

**DOI:** 10.1038/s41419-018-0809-1

**Published:** 2018-07-10

**Authors:** Feifeng Song, Yaodong Yi, Cui Li, Yongjun Hu, Jinhai Wang, David E. Smith, Huidi Jiang

**Affiliations:** 1 Laboratory of Pharmaceutical Analysis and Drug Metabolism, Zhejiang Province Key Laboratory of Anti-Cancer Drug Research, College of Pharmaceutical Sciences, Zhejiang University, Hangzhou, 310058 Zhejiang China; 2 Department of Pharmaceutical Sciences, College of Pharmacy, University of Michigan, Ann Arbor, Michigan 48109 United States; 3 The First Affiliated Hospital, College of Medicine, Zhejiang University, 79 Qingchun Road, Hangzhou, 310003 Zhejiang China

## Abstract

The peptide/histidine transporter SLC15A3 is responsible for transporting histidine, certain dipeptide and peptidomimetics from inside the lysosome to cytosol. Previous studies have indicated that SLC15A3 transcripts are mainly expressed in the lymphatic system, however, its regulation and biological role in innate immune responses and inflammatory diseases are as yet unknown. In this study, mouse peritoneal macrophages (PMs), mouse bone marrow-derived macrophages (BMDMs), the human acute monocytic leukemia cell line THP-1 and the human lung epithelial carcinoma cell line A549 were used to investigate the regulation and biological role of SLC15A3 in TLR-mediated inflammatory responses. Our results showed that SLC15A3 was upregulated by TLR2, TLR4, TLR7 and TLR9 ligands in macrophages at both the mRNA and protein levels *via* activation of NF-κB (nuclear factor-kappa-B), MAPK (mitogen-activated protein kinase) and IRF3 (interferon regulatory factor 3). Furthermore, knockdown or overexpression of SLC15A3 influenced the TLR4-triggered expression of proinflammatory cytokines. A reporter gene assay showed that the SLC15A3 promotor contained potential NF-κB binding sites, which were reasonable for regulating SLC15A3 by TLR-activation through NF-κB signaling. Additionally, SLC15A3 expression was increased and positively related to inflammation in mice with bacterial peritonitis. The collective findings suggest that SLC15A3 is regulated by various TLRs, and that it plays an important role in regulating TLR4-mediated inflammatory responses.

## Introduction

Solute carrier (SLC) 15A3, a member of the proton-coupled oligopeptide transporter (POT) family, is responsible for translocating certain dipeptide and histidine across biological membranes^1^. Two POT family members, SLC15A1 (PEPT1) and SLC15A2 (PEPT2) possess around 50% amino acid identity, and their respective physiological roles in the small intestine and kidney have been well characterized. In contrast, SLC15A3 (PHT2) and SLC15A4 (PHT1) have poor amino acid homology with SLC15A1 and SLC15A2 (<20%), and their substrate specificity, transport kinetics, and pharmacologic relevance are largely unknown. SLC15A3 is an endosomal and lysosomal transporter, which is mainly expressed in the lung, spleen and thymus^1–3^. Recent studies showed that SLC15A3 mRNA expression was increased by the TLR4 (Toll-like receptor 4) agonist lipopolysaccharide (LPS) in mouse bone marrow-derived dendritic cells (BMDCs), and the macrophage cell lines J774A.1 and THP-1^2,4^. Nakamura et al. also found that the production of interleukin 6 (IL-6) and interleukin 1β (IL-1β) in *SLC15A3*-deficient dendritic cells was significantly lower than wild-type cells when stimulated with muramyl dipeptide (MDP), a NOD2 (nucleotide-binding oligomerization domain 2) ligand^2^. However, the molecular regulatory mechanism and biological role of SLC15A3 in TLR-mediated inflammatory responses remain poorly understood.

Inflammation is a response of the innate immune system to pathogens or injury, and can be induced through activation of TLRs by pathogen-associated molecular patterns (PAMPs) such as acylated lipoproteins (TLR2 ligand), LPS (TLR4 ligand), signal stranded RNA (ssRNA, TLR7/8 ligand) and unmethylated DNA (TLR9 ligand)^5,6^. The interaction of TLR and PAMP triggers several signaling cascades that leads to the production of inflammatory cytokines and type I interferons (IFNs)^5^. All TLRs except TLR3 can transmit signals *via* the myeloid differentiation factor 88 (MyD88)-dependent pathway, which activate nuclear factor kappa B (NF-κB) and mitogen-activated protein kinases (MAPKs), resulting in the transcription of many proinflammatory genes. TLR4 also triggers the TIR domain-containing adaptor protein (TIRAP) which induces interferon β (TRIF)-dependent signaling and the activation of interferon regulatory factor 3 (IRF3), thereby, resulting in IFN-α and IFN-β gene transcription^7^. These TLR-induced inflammatory mediators are required for pathogen clearance, but many mediators are toxic to the host and their exaggerated production can further augment systemic inflammation. It has been demonstrated that TLR-induced cytokines are implicated in several autoimmune diseases, such as rheumatoid arthritis, inflammatory bowel disease, and systemic lupus erythematosus^8^. Because TLR-induced mediators perform both protective and toxic functions, it is important to better understand the TLR signaling pathway and to dampen the production of proinflammatory mediators.

SLC15A4, another POT family member, is also localized in endosomes and lysosomes, and has similar transport properties with SLC15A3^9,10^. Several studies have reported that SLC15A4 is closely associated with inflammatory diseases such as diabetes, systemic lupus erythematosus and inflammatory bowel disease^11–14^. In *SLC15A4*-deficient plasmacytoid dendritic cells (pDCs), TLR7 and TLR9-induced type I IFNs and proinflammatory cytokines production were abrogated^13,15^. Studies have reported that SLC15A4 is essential for signaling through TLR7 and TLR9, but it is unclear whether SLC15A3 also has a role in TLR7 and TLR9 signaling. Currently, both SLC15A4 and TLRs are associated with some inflammatory diseases, but limited information is available concerning the relationship of SLC15A3 with inflammatory diseases.

With this in mind, the aim of the present study was to explore the regulation and biological role of SLC15A3 in the TLR-mediated inflammatory responses of mouse primary peritoneal macrophages (PMs) and bone marrow-derived macrophages (BMDMs), human acute monocytic leukemia cell line THP-1, and the human lung epithelial carcinoma cell line A549. We also investigated whether SLC15A3 was linked to inflammatory diseases by using a mouse model of peritonitis. Collectively, the proposed studies will help to elucidate the molecular regulatory mechanism of SLC15A3 in inflammatory responses and immune diseases, and to better understand the role of SLC15A3 in immune diseases.

## Results

### SLC15A3 was preferentially expressed in macrophages and upregulated by TLR4 activation

**Fig. 1:**
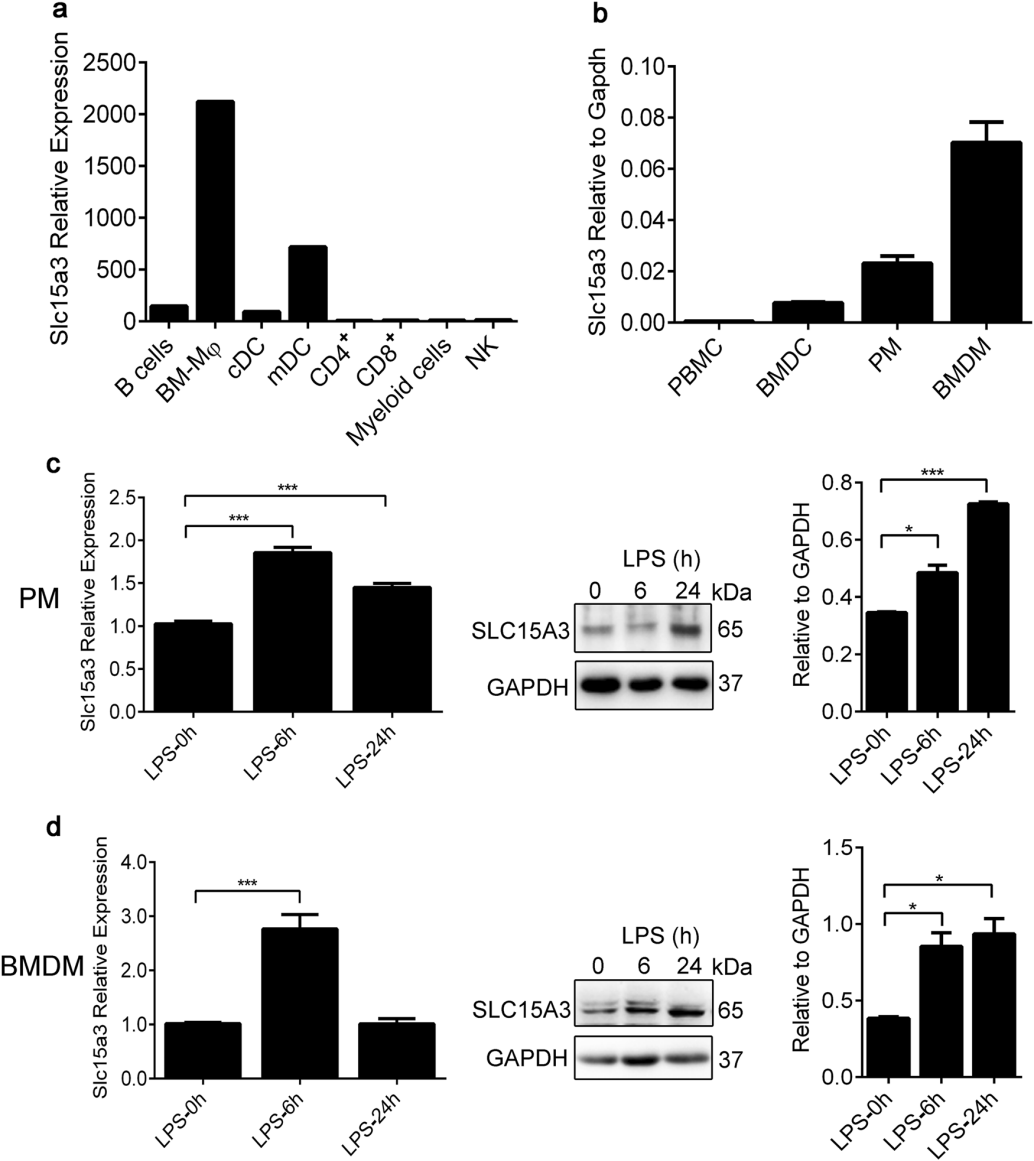
SLC15A3 was preferentially expressed in macrophages and upregulated by TLR4 activation. **a** The expression profile of Slc15a3 in mouse leukocytes based on cDNA data. **b** mRNA expression of Slc15a3 in mouse PBMCs, BMDCs, PMs and BMDMs isolated from ICR mice. **c**, **d** mRNA and protein expression of Slc15a3 in mouse PMs (**c**) and BMDMs (**d**) treated with 100 ng/mL LPS for specified periods of time. Quantification of protein (i.e., SLC15A3/GAPDH ratio) is shown in the right side of each Western blot figure. One-way ANOVA followed by Dunnett’s test was used to evaluate the statistical differences. ^*^P *<* 0.05, ^**^P < 0.01, and ^***^P *<* 0.001 compared with 0 h. The data are expressed as mean ± SE (*n* = 3)

On the basis of cDNA gene expression analysis, we found that Slc15a3 was expressed at a much higher level in macrophages and dendritic cells than in other mouse peripheral blood leukocytes (Fig. 1a). To confirm this result, peripheral blood mononuclear cells (PBMCs), BMDCs, PMs and BMDMs were isolated from mice, and Slc15a3 transcriptional levels were quantified by real-time PCR. As shown in Fig. 1b, the Slc15a3 transcript was present in macrophages and BMDCs, the levels of Slc15a3 were about 125-fold, 40-fold and 14-fold higher, respectively, in BMDMs, PMs and BMDCs as compared to PBMCs.

Macrophages play a crucial role in pathogen recognition and the clearance of bacteria and bacterial components, as they are equipped with a wide range of pattern recognition receptors^16^. Since Slc15a3 is highly expressed in macrophages and responsible for transporting some bacterial components across biological membranes^17^, we investigated whether Slc15a3 could be regulated by TLR4 activation. As shown in Fig. 1c, d, Slc15a3 was significantly upregulated in LPS-treated mouse PMs and BMDMs at both the mRNA and protein levels.

### SLC15A3 was upregulated by activation of NF-κB and the promoter region of SLC15A3 contained NF-κB binding sites

**Fig. 2:**
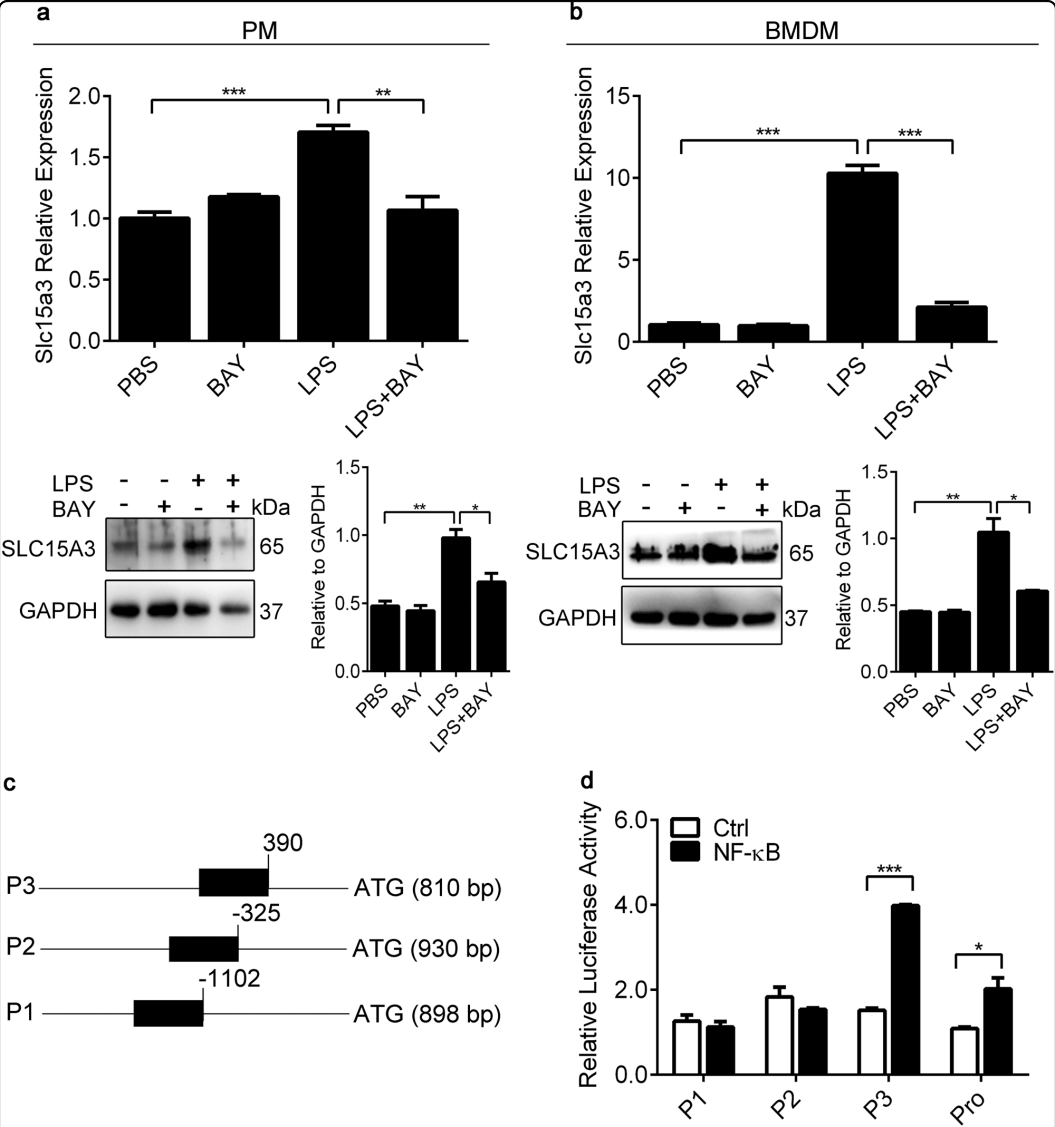
SLC15A3 was regulated by the activation of NF-κB and the promotor region of SLC15A3 contained potential NF-κB binding sites. **a**, **b** mRNA and protein expression of Slc15a3 in mouse PMs (**a**) and BMDMs (**b**) pretreated with or without 10 μM BAY 11–7082 (BAY) for 1 h, and then treated with or without 100 ng/mL LPS for another 4 h or 24 h for mRNA and protein detection, respectively. Quantification of protein (i.e., SLC15A3/GAPDH ratio) is shown in the right side of each western blot figure. One-way ANOVA followed by Tukey’s test was used to evaluate the statistical differences, ^*^P *<* 0.05, ^**^P < 0.01 and ^***^P *<* 0.001. **c** Mouse Slc15a3 promotor constructs of varying length were fused to the luciferase reporter gene in the pGL3-basic vector. Predicted binding sites are shown schematically (black boxes), where the binding site numbers indicate position 5′ upstream of the start codon. **d** Luciferase activity in HeLa cells transfected with luciferase reporter constructs (horizontal line), with or without pcDNA3.1-mNF-κB, were normalized to renilla luciferase activity and presented relative to those transfected with pcDNA3.1 cells. Statistical analyses were performed using an unpaired *t* test. ^*^P *<* 0.05, ^**^P < 0.01, and ^***^P *<* 0.001 compared with the vehicle group. The data are expressed as mean ± SE (*n* = 3)

Because LPS activates TLR4 and subsequently leads to the activation of NF-κB, we next determined whether NF-κB activation was required for the LPS-induced upregulation of Slc15a3 in mouse PMs and BMDMs. As shown in Fig. 2a, b, the upregulation of Slc15a3 was abolished by the specific NF-κB inhibitor BAY 11–7082. Moreover, TLR4 signaling induced the transcription of Il-6 and Tnf-α, which were also reduced by BAY 11–7082 (Supplementary Figure S1). The expression changes of Slc15a3 displayed in a similar trend as proinflammatory cytokines (e.g. Il-6, Tnf-α), indicating that Slc15a3 might paly a role in the TLR4 signal pathway.

Since Slc15a3 could be upregulated by LPS (NF-κB activation) and suppressed by BAY 11–7082 (NF-κB inactivation), it is reasonable to infer that NF-κB might directly regulate Slc15a3 expression. Therefore, we applied a reporter gene assay to evaluate whether the promoter region of Slc15a3 contained potential NF-κB binding sites. As shown in Fig. 2d, the 2.5 kb region of mouse Slc15a3 promotor induced a 2-fold increase in luciferase activity upon NF-κB co-transfection as compared to vehicle. Shorter promotor constructs were then generated to analyze the functional role of the three putative mouse Slc15a3 binding sites in response to NF-κB co-transfection. The constructs containing NF-κB binding sites had lengths of 810 bp (P3), 930 bp (P2) and 898 bp (P1) (Fig. 2c). Only P3, the construct of 810 bp length, showed a significant response to NF-κB overexpression (Fig. 2d). These results indicate that a functional binding site exists in the mouse Slc15a3 promotor, which is essential to activate the NF-κB response.

### Blocking MAPK and TRIF signal pathway diminished LPS-induced SLC15A3 upregulation

**Fig. 3:**
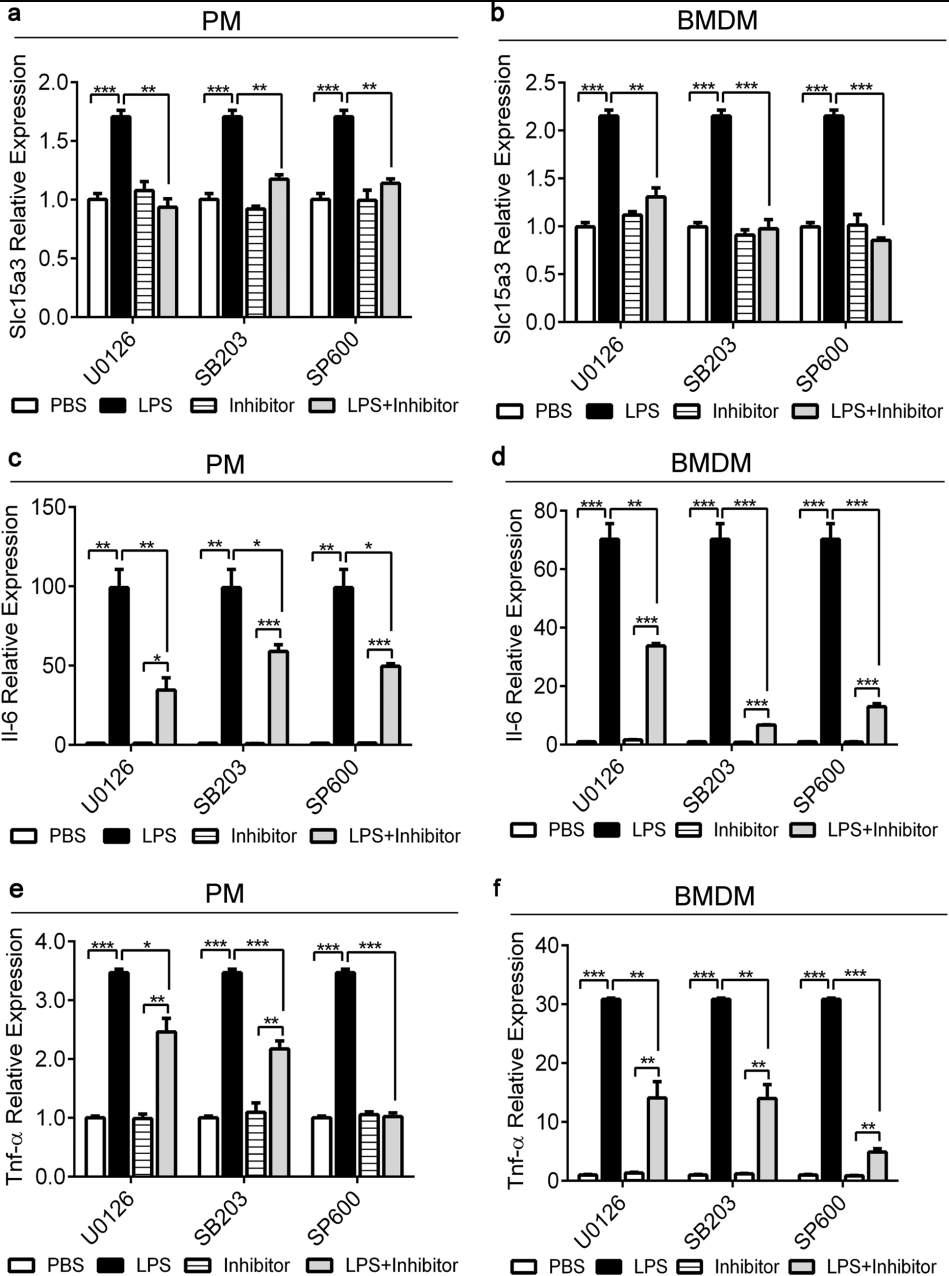
Blocking the MAPK signaling pathway diminished LPS-induced SLC15A3 upregulation. Mouse PMs (left panel) and BMDMs (right panel) were pretreated with or without 50 μM U0126, 10 μM SB203085 (SB203) and 10 μM SP600125 (SP600) for 1 h, then the cells were treated with or without 100 ng/mL LPS for another 4 h. mRNA expression of Slc15a3 (**a**, **b**), Il-6 (**c**, **d**) and Tnf-α (**e**, **f**) were determined by real-time PCR. One-way ANOVA followed by Tukey’s test was used to evaluate the statistical differences, ^*^P *<* 0.05, ^**^P < 0.01, and ^***^P *<* 0.001. The data are expressed as mean ± SE (*n* = 3)

TLR4 requires the adaptors MyD88 and TRIF to effectively activate NF-κB and MAPK, resulting in the transcription of various proinflammatory cytokine genes. As NF-κB activation was responsible for the LPS-induced expression of Slc15a3 (Fig. 2), whether MAPK activation contributed to LPS-induced Slc15a3 needs to be further characterized. We detected the expression of Slc15a3 in mouse PMs and BMDMs treated with or without specific MAPK inhibitors, such as the MEK1/2 inhibitor U0126, the p38 inhibitor SB203580 and the JNK inhibitor SP600125. Our data revealed that the LPS-induced upregulation of Slc15a3 was abrogated by the presence of 50 μM U0126, 10 μM SB203580 and 10 μM SP600125 (Fig. 3a, b), as was the LPS-induced expression of Il-6 and Tnf-α in PMs and BMDMs (Fig. 3c–f).

**Fig. 4:**
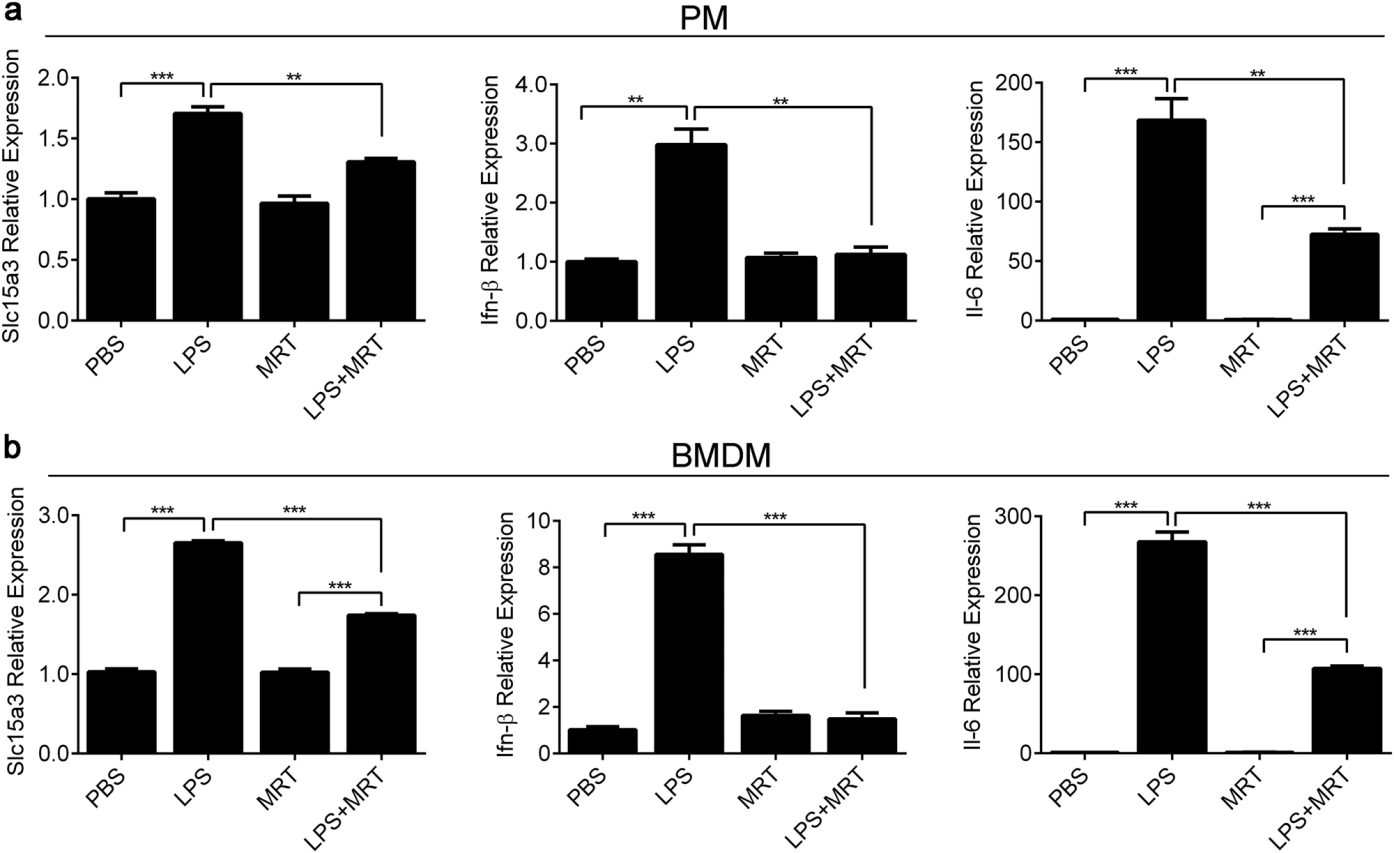
TRIF inhibitor decreased LPS-induced SLC15A3 upregulation. **a** and **b** Mouse PMs (**a**) and BMDMs (**b**) were pretreated with or without 2 μM MRT67307 (MRT) for 1 h, then the cells were treated with or without 100 ng/mL LPS for 4 h. mRNA expression of Slc15a3, Ifn-β and Il-6 were determined by real-time PCR. One-way ANOVA followed by Tukey’s test was used to evaluate the statistical differences, ^*^P *<* 0.05, ^**^P < 0.01 and ^***^P *<* 0.001. The data are expressed as mean ± SE (*n* = 3)

TLR4 can transmit signals in a TRIF-dependent pathway that also activates IRF3, which leads to the production of type I IFNs. MRT67307 (a novel inhibitor of TRIF signaling), by blocking the activation of IKKε/TBK1 (IKK-related kinases/TANK-binding kinase 1), suppresses the production of IFN-β^18^. Here we treated mouse PMs and BMDMs with or without 2 μM MRT67307, and then examined Slc15a3 and Ifn-β expression. As shown in Fig. 4, MRT67307 significantly attenuated the LPS-induced expression of Slc15a3, Ifn-β and Il-6 in PMs and BMDMs.

### SLC15A3 expression was also regulated by TLR2, TLR7 and TLR9 ligands

**Fig. 5:**
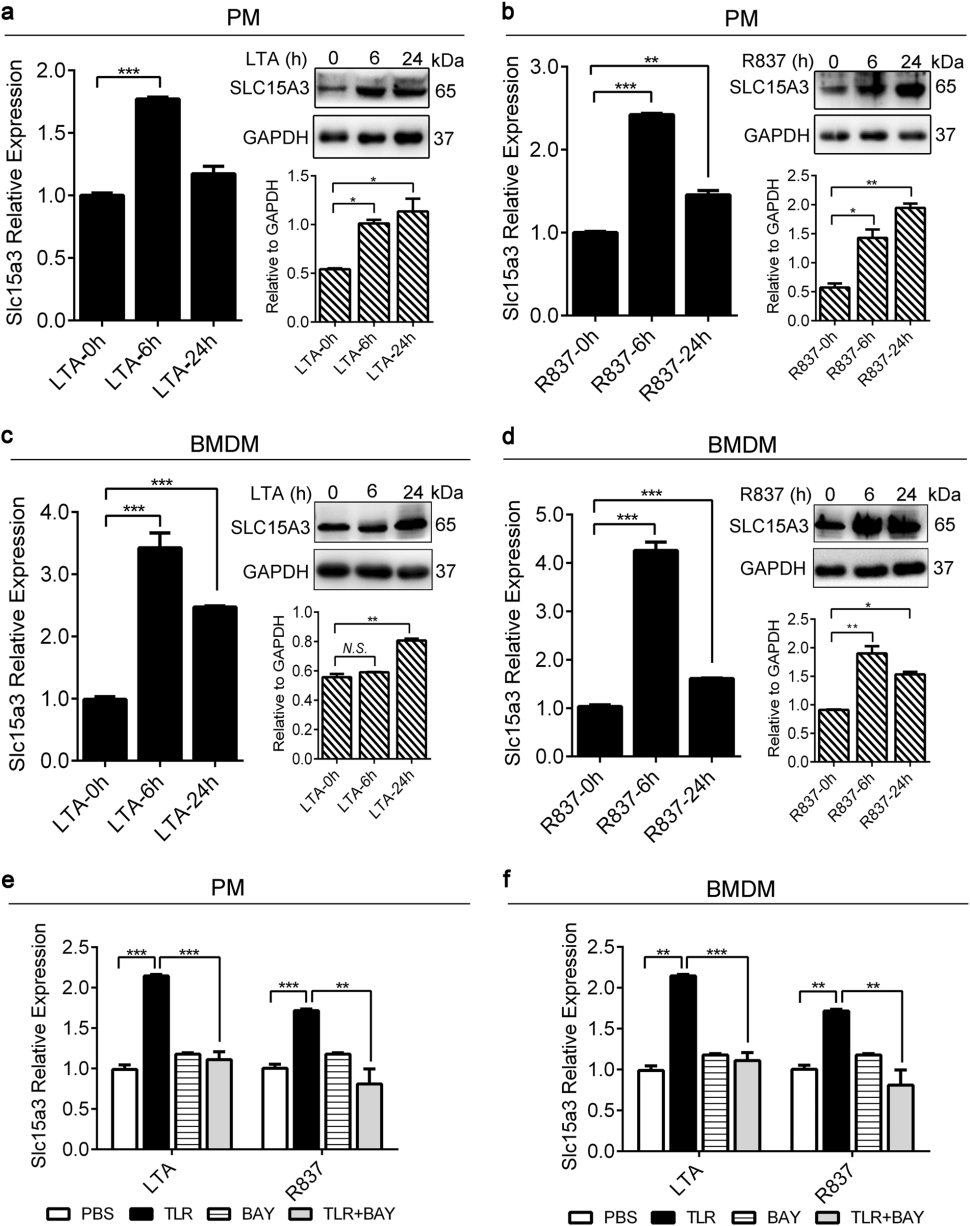
SLC15A3 expression was upregulated by TLR2 and TLR7 ligands. **a**–**d** mRNA and protein expression of Slc15a3 in mouse PMs (**a**, **b**) and BMDMs (**c**, **d**) treated with 1 μg/mL LTA or 5 μg/mL R837 for specified periods of time. Quantification of protein (i.e., SLC15A3/GAPDH ratio) is shown below of each Western blot figure. One-way ANOVA followed by Dunnett’s test was used to evaluate the statistical differences. ^*^P < 0.05, ^**^P < 0.01, and ^***^P < 0.001 compared with 0 h. **e**, **f** Mouse PMs (**e**) and BMDMs (**f**) were pretreated with or without 10 μM BAY 11–7082 (BAY) for 1 h, then treated with or without 1 μg/mL LTA or 5 μg/mL R837 for 4 h. mRNA expression of Slc15a3 were determined by real-time PCR. One-way ANOVA followed by Tukey’s test was used to evaluate the statistical differences, ^*^P < 0.05, ^**^P < 0.01 and ^***^P *<* 0.001. The data are expressed as mean ± SE (*n* = 3)

As Slc15a3 was upregulated by TLR4 activation, we speculated that other TLRs might also be involved in regulating Slc15a3 expression. Our results revealed that the mRNA and protein expression of Slc15a3 in mouse PMs and BMDMs were markedly induced by 1 μg/mL LTA (TLR2 agonist) and 5 μg/mL R837 (TLR7 agonist) (Fig. 5a–d), as well as by 1 μM CpG A or 0.2 μM CpG B (TLR9 agonist) (Supplementary Figure S2), indicating that Slc15a3 was also upregulated *via* activation of TLR2, TLR7 and TLR9.

TLRs 2, 7 and 9 all require MyD88 to activate NF-κB, and Slc15a3 has NF-κB binding sites in its promoter region. Thus, inhibition of NF-κB might influence the expression of Slc15a3 by these TLRs. Our data showed that the LTA- (TLR2 agonist) and R837 (TLR7 agonist)-induced upregulation of Slc15a3, Il-6 and Tnf-α were suppressed in mouse PMs and BMDMs pretreated with 10 μM BAY 11–7082 (NF-κB inhibitor) (Fig. 5e, f, Supplementary Figure S1). These findings indicate that TLR2 and TLR7, along with NF-κB activation, are involved in the regulation of Slc15a3 and downstream cytokine responses. Like-minded studies were not performed for CpG A (or CpG B), but it is likely that similar results would be observed for TLR9.

### Knockdown or overexpression of SLC15A3 influenced TLR4-dependent proinflammatory cytokine production

**Fig. 6:**
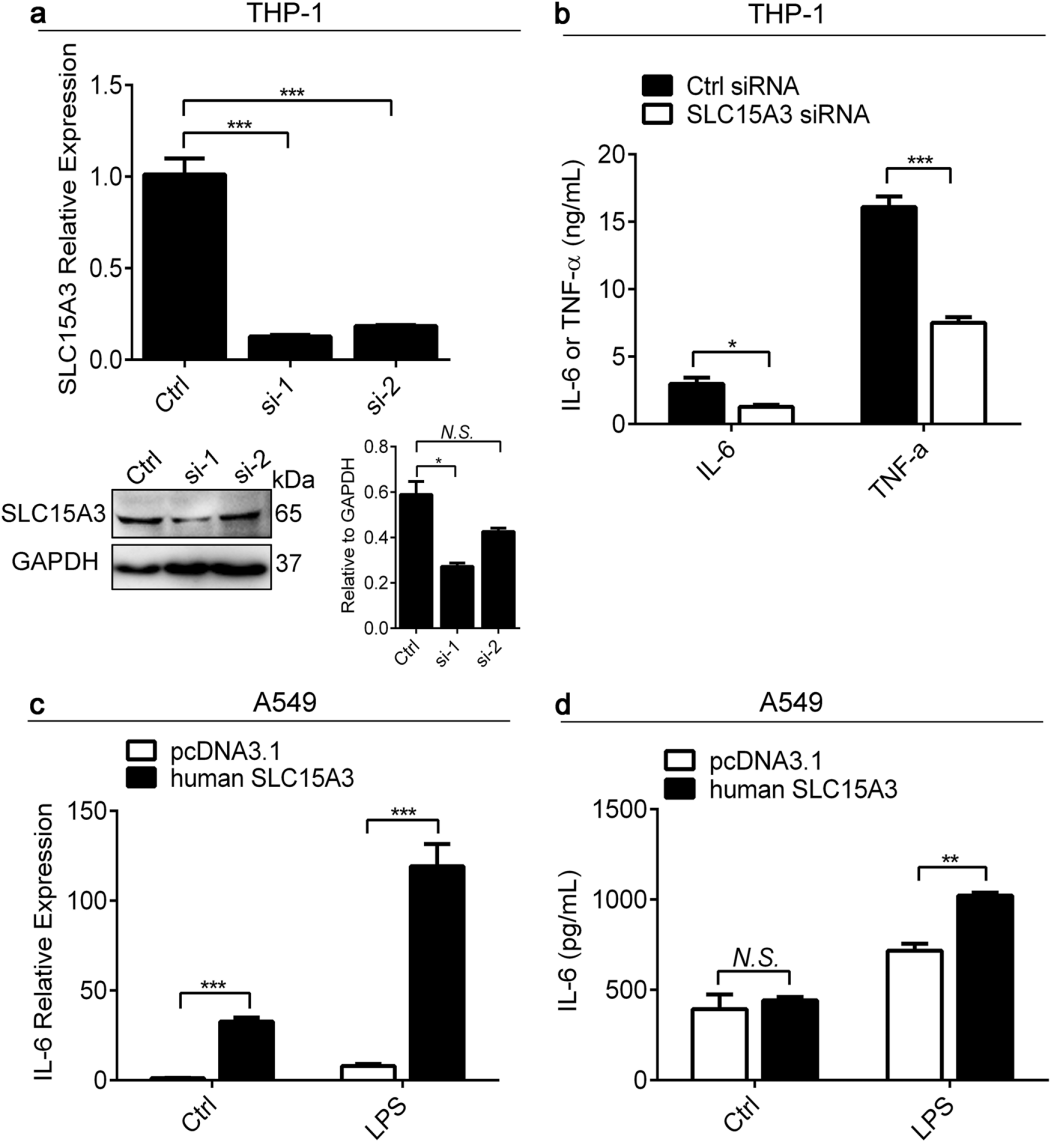
Knockdown or overexpression of SLC15A3 influenced the TLR4-dependent proinflammatory cytokine production. **a** mRNA and protein expression of SLC15A3 was determined in THP-1 cells transfected with 30 nM control siRNA (Ctrl) or two different SLC15A3 siRNA (#1 and #2) after 24 h transfection. Quantification of protein (i.e., SLC15A3/GAPDH ratio) is shown in the right side of each Western blot figure. **b** THP-1 cells transfected with 30 nM control siRNA (Ctrl) or SLC15A3 siRNA (#1). Twenty-four hours after transfection, the cells were treated with 100 ng/mL LPS for 12 h. The levels of IL-6 and TNF-α in the cell culture supernatants were determined by ELISA assays. **c**, **d** A549 cells transfected with 1 μg pcDNA3.1 or human SLC15A3 plasmid. Twenty-four hours after transfection, the cells were treated with 1 μg/mL LPS for 8 h. The mRNA (**c**) and protein (**d**) levels of IL-6 in cells and cell culture supernatants were determined by real-time PCR and ELISA assays. Statistical analyses were performed using an unpaired *t* test. ^*^P *<* 0.05, ^**^P < 0.01, and ^***^P *<* 0.001 compared with control (Ctrl). The data are expressed as mean ± SE (*n* = 3)

On the basis of our previous findings in mice, we hypothesized that SLC15A3 might play a role in the inflammatory responses of macrophages to TLR4 stimulation in humans. To test this hypothesis, we first compared the LPS-induced production of inflammatory cytokines in normal and SLC15A3 knockdown THP-1 cells. THP-1 cells were treated with two different SLC15A3 siRNAs, and then SLC15A3 was quantified at the transcript and protein levels. As shown in Fig. 6a, both siRNAs effectively knocked down the SLC15A3 mRNA (by approximately 90%) but only siRNA (#1) knocked down the SLC15A3 protein (about 50%). Moreover, the LPS-induced upregulation of IL-6 and TNF-α was significantly inhibited (*P* < 0.05, 0.001, respectively) in SLC15A3 knockdown cells, as compared to normal cells (Fig. 6b). We also overexpressed SLC15A3 in A549 cells and found that overexpression of this transporter significantly enhanced LPS-triggered IL-6 production (*P* < 0.01) (Fig. 6c, d). The data suggest that SLC15A3 may play a role in regulating TLR-dependent proinflammatory cytokines production in human macrophages and other tissues such as lung.

### Knockdown of SLC15A3 did not affect LPS-induced proximal cytoplasmic signaling

**Fig. 7:**
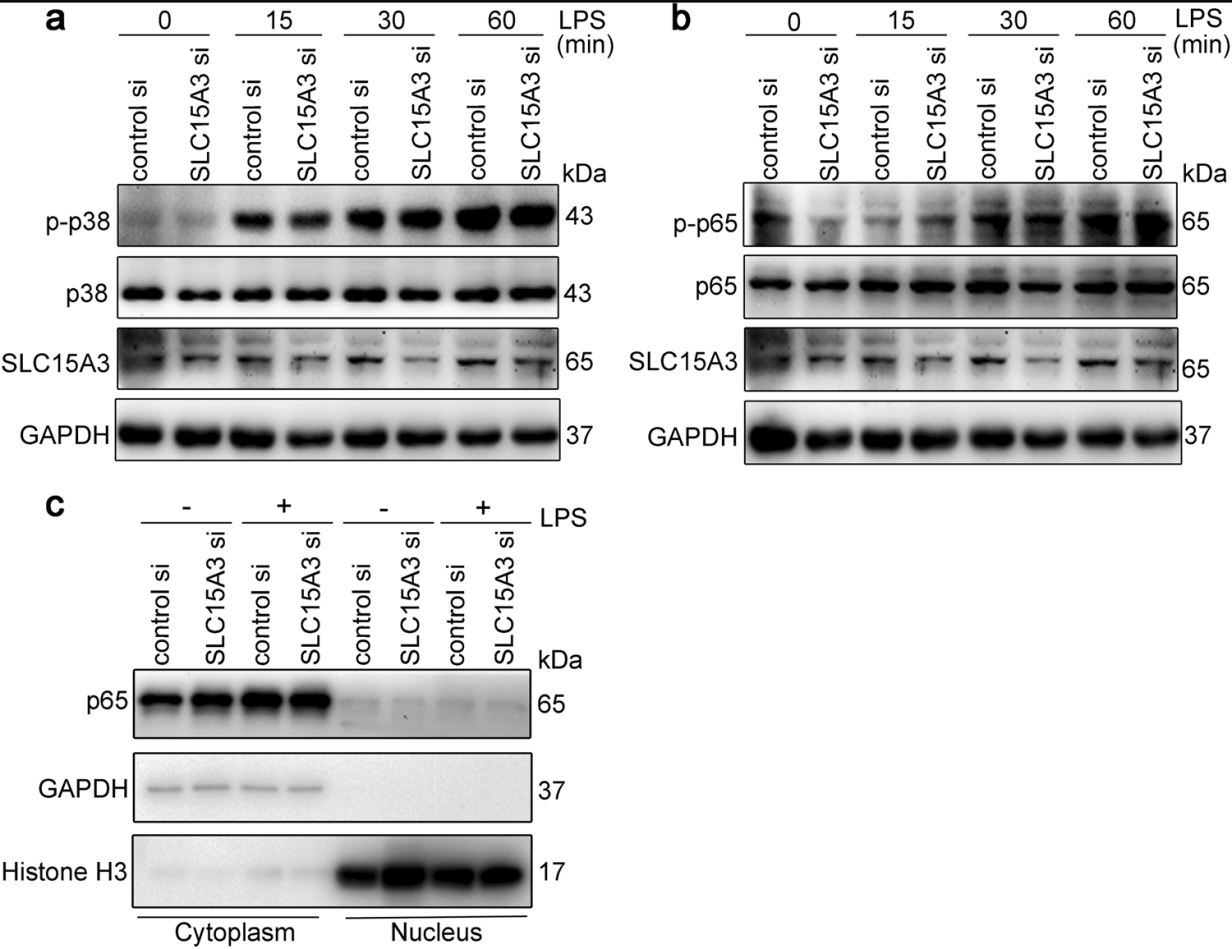
Knockdown of SLC15A3 did not affect the proximal cytoplasmic signaling. **a**, **b** Protein expression of p-p38, p38, p-p65, p65, SLC15A3 and GAPDH in THP-1 cells transfected with 30 nM control siRNA or SLC15A3 siRNA. Twenty-four hours after transfection, the cells were treated with 100 ng/mL LPS for the specified period of time. **c** Protein expression of p65, histone H3 and GAPDH in THP-1 cells transfected with 30 nM control siRNA or SLC15A3 siRNA. Twenty-four hours after transfection, the cells were treated with 100 ng/mL LPS for 30 min, and then separated into cytoplasmic and nuclear fractions for the determination of p65 protein expression. The purity of cytoplasmic and nuclear fractions was confirmed by cytoplasmic (GAPDH) and nuclear (histone H3) markers

The results above showed that knockdown of SLC15A3 significantly attenuated the LPS-induced expression of IL-6 and TNF-α. To further delineate the mechanism, we examined the proximal cytoplasmic signaling events upon LPS stimulation. As shown in Fig. 7a,b, knockdown of SLC15A3 had no effect on LPS-induced p38 or p65 phosphorylation. Moreover, there was no difference in translocation of the NF-κB subunit p65 into nuclei of LPS-treated THP-1 cells with knockdown of SLC15A3 (Fig. 7c). These data suggest that SLC15A3 does not participate in LPS-induced cytoplasmic signaling events.

### SLC15A3 expression was increased in mice with *E. coli*-induced peritonitis and positively related to inflammation

**Fig. 8:**
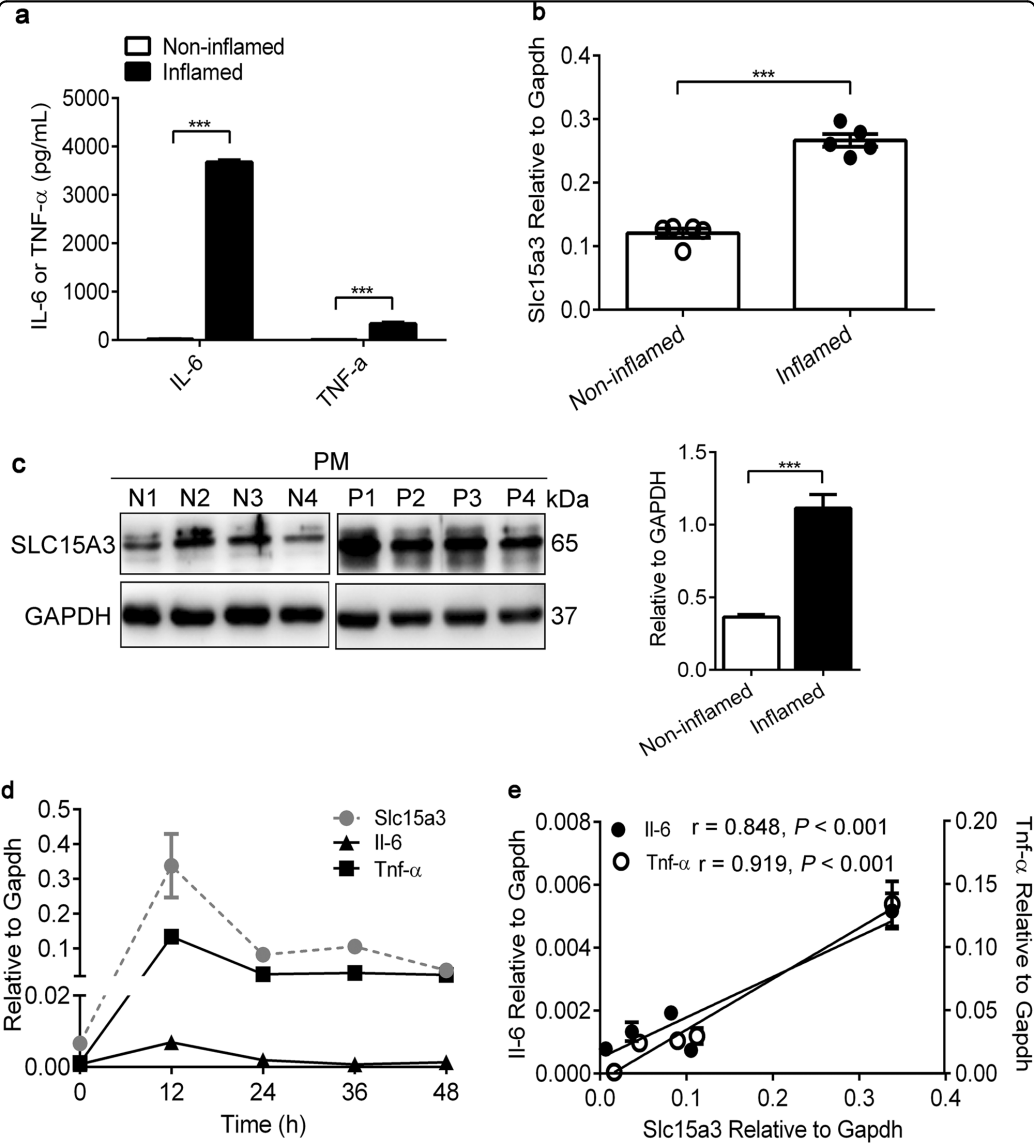
SLC15A3 expression was increased in mice with *E. coli*-induced peritonitis, and positively related to inflammation. Mice were intraperitoneally injected with PBS or *E.coli* (1 × 10^7^ cfu/mL, 0.5 mL/ 25 g), and the serum or peritoneal macrophages collected after 24 h. **a** IL-6 and TNF-α protein levels in mouse serum were determined by ELISA assays. **b**, **c** Slc15a3 mRNA (**b**) and protein expression (**c**) in PMs was determined by real-time PCR and Western blotting. Quantification of protein (i.e., SLC15A3/GAPDH ratio) is shown in the right side of each western blot figure. **d** mRNA expression of Il-6 and Tnf-α in peritoneal macrophages as a function of time. **e** Correlation analysis of Slc15a3 with Il-6 or Tnf-α was performed by linear regression, in which Slc15a3 mRNA expression in x-axis and Il-6 (left) or Tnf-α (right) mRNA expression in *y*-axis. Statistical analyses were performed using an unpaired *t* test. ^*^P *<* 0.05, ^**^P < 0.01, and ^***^P *<* 0.001 compared with PBS group. The data are expressed as mean ± SE (*n* = 4–6)

Studies have linked some inflammatory diseases to the alteration of SLC15A4 expression. Because of the similarity between SLC15A3 and SLC15A4, we aimed to identify whether the expression of SLC15A3 was changed in mice with peritonitis induced by *E.coli*. As shown in Fig. 8a, serum IL-6 and TNF-α levels were significantly higher in *E. coli* treated mice as compared to vehicle treated mice (*P* < 0.001), indicating that peritonitis was successfully induced. Concomitantly, the expression of Slc15a3 in PMs separated from mice with peritonitis was markedly upregulated at both the mRNA and protein levels as compared to non-inflamed mice (Fig. 8b, c). Finally, the alteration of Slc15a3 expression was positively related to proinflammatory cytokines in mice with peritonitis stimulated by *E. coli* as a function of time (Fig. 8d, e).

## Discussion

There is a paucity of the data on the regulation and biological role of SLC15A3 in innate immune responses, such as TLR and NOD-mediated signaling. Previous immunofluorescence and uptake experiments in mouse BMDCs have demonstrated that SLC15A3 was required for NOD2 in response to endosomal derived MDP^2^. Moreover, the transcriptional level of SLC15A3 was induced in TLR3 and TLR4-stimulated mouse microglial BV-2 cells^19^, indicating that SLC15A3 plays an important role in innate immune responses. However, these latter studies did not evaluate the regulatory mechanism and role of SLC15A3 in TLR-triggered immune responses.

In the present study, we reported several novel findings regarding the regulation and biological role of SLC15A3 in TLR-mediated inflammatory responses. We found that: (1) SLC15A3 was upregulated in vitro by the activation of TLR2, TLR4, TLR7 and TLR9 in macrophages and increased in PMs from mice with peritonitis; (2) the increased expression of SLC15A3 by TLR ligands could be attenuated by the inhibitors of NF-κB, MAPK and TRIF, and the SLC15A3 promotor region contained potential NF-κB binding sites; (3) knockdown or overexpression of SLC15A3 influenced TLR4-triggered proinflammatory cytokine production, but did not affect TLR4 agonist-induced proximal cytoplasmic signaling; and (4) SLC15A3 expression was positively related to inflammation in mice with peritonitis. Taken together, our study revealed that SLC15A3 could be regulated by activation of TLRs *via* NF-κB, MAPK and TRIF signaling and, in turn, might regulate the TLR4 signaling pathway.

SLC15A3, one of several lysosomal membrane transporters, mediates the intracellular sensing of microbial pathogens in dendritic cells^2^. In fact, more than 25 integral lysosomal membrane proteins play critical roles in maintaining the morphology and function of lysosomes in mammals, such as SCARB2 (scavenger receptor class B, member 2)^20–22^. It has been reported that SCARB2 could be upregulated by TLR9 agonists and that its knockdown greatly diminished the TLR9-induced production of IFN-α and IFN-β in pDCs^23^. Similar to SCARB2, our data showed that SLC15A3 was induced by various TLR ligands, and knockdown of SLC15A3 decreased the TLR4-induced production of IL-6 and TNF-α in macrophages (Supplementary Figure S3). These results indicate that SLC15A3 might be an important protein for maintaining the morphology and function of endosomes and lysosomes, and that its overexpression or knockdown might lead to dysregulation of endocytic membrane trafficking.

SLC15A3 is targeted to endosomal and lysosomal membranes, which is mediated by a di-leucine-based motif “GERQPLL” in the N-terminal cytoplasmic domain^1,2^. This motif is known to be required for binding to AP-3, an adaptor protein complex that transports proteins from late Golgi to the vacuole^24^. It was found that AP-3 and SLC15A4 were required for TLR7 and TLR9 signaling in pDCs^25^. In agreement with the above findings, our results showed that SLC15A3 could be induced by TLR7 and TLR9 ligands in macrophages (Fig. 5 and Supplementary Figure S2), indicating that the sorting of SLC15A3 might be associated with AP-3. The molecular mechanism of this interaction, however, needs to be further studied.

Several studies have shown that transcriptional factors have a significant effect on intestinal SLC15A1 expression and activity. For example, the transcriptional factors Sp1, Cdx2 and PPARα were shown to be responsible for the basal, intestine-specific expression of SLC15A1^26–28^. An in vitro study also found that the human SLC15A1 promoter contained some Nrf2 binding sites^29^. Our reporter gene assay demonstrated that the mouse SLC15A3 promotor had NF-κB binding sites (Fig. 2), which was consistent with results showing that inhibition of NF-κB led to downregulation of SLC15A3 expression (Fig. 2 and Fig. 5). However, little information is available about the possible effects of transcriptional factors (e.g., Sp1, Cdx2, PPARα, Nrf2) on SLC15A3 regulation.

To investigate the role of SLC15A3 in TLR4-mediated inflammatory responses, we tried to knock down Slc15a3 in mouse primary macrophages by transducing siRNA or shRNA into the cells using Lipofectamin or jetPrime. However, these attempts were unsuccessful (data not shown), perhaps due to the low transfection efficiency or the unsuitable siRNA or shRNA for mouse SLC15A3. Because of the technical challenges to knock down SLC15A3 in mouse primary macrophages, we adopted the human cell line THP-1 for further study (Fig. 6). Since knockdown of SLC15A3 decreased the expression of inflammatory cytokines, we then tried to verify whether overexpression of SLC15A3 increased the production of cytokines. We screened several human cell lines (e.g., HeLa, HepG2, Huh-7, HL-7702, DU-145, PC-3, BCap37) but, ultimately, found one that was suitable, A549. This cell line had high transfection efficiency, low endogenous SLC15A3 expression, and its cell membrane expressed the TLR4 receptor.

Spontaneous bacterial peritonitis (SBP) is a frequent complication in cirrhotic and ascitic patients, whom have a poor prognosis and high mortality rate^30,31^. During bacterial infection in the abdominal cavity, monocytes and macrophages are crucial for pathogen recognition and the clearance of bacteria and bacterial components, as they express a wide range of pattern recognition receptors (PRRs) for PAMP recognition, thereby, inducing the production of inflammatory cytokines^32^. Studies have found that genetic variants of PRRs such as *TLR2* and *NOD2* were associated with the development of SBP^33–35^. Moreover, TLR4 has been suggested as a potential therapeutic target for intraabdominal infection since the yield of Gram-negative bacteria was much lower in TLR4 mutants than wild-type mice^36^. On the basis of the finding that knockdown of SLC15A3 decreased TLR4-dependent IL-6 and TNF-α production (Fig. 6), SLC15A3 was found to increase in mice with peritonitis, which was positively related to the development of inflammation (Fig. 8). From these results, we deduced that SLC15A3 might be a therapeutic target in inflammatory diseases such as bacterial peritonitis.

Taken together, our study revealed that SLC15A3 could be regulated by various TLRs via activation of MyD88/NK-κB, MyD88/MAPK and TRIF/IRF3 signaling. Moreover, SLC15A3 also regulated TLR4-dependent proinflammatory cytokine production and was positively related to inflammation in mice with bacterial peritonitis. These findings suggest that SLC15A3 might play an important role in TLR-mediated inflammatory responses.

## Materials and methods

### Animals and cells

Male (6–8 weeks) ICR mice used in the present study were purchased from Shanghai SLRC Laboratory Animal of the Chinese Academy of Sciences (Shanghai, China). Animals were housed in Zhejiang University Laboratory Animal Center, and were allowed free access to standard laboratory chow and tap water prior to study. Animal studies were approved by the review committee of Zhejiang University, College of Pharmaceutical Sciences, and were in compliance with institutional guidelines.

The human acute monocytic leukemia cell line THP-1, HeLa and A549 cells were kindly provided by Dr. Dajing Xia, Dr. Shuqing Chen and Dr. Weimin Fan, respectively (Zhejiang University, Hangzhou). All cell lines were cultured in Roswell Park Memorial Institute 1640 (RPMI-1640) from Invitrogen (Rockford, IL) supplemented with 10% Fetal bovine serum (FBS) from ThermoFisher Scientific (Rockford, IL) and 1% penicillin/streptomycin in a 5% CO_2_ incubator at 37 °C.

The cDNA data were available in BioGPS database (http://biogps.org) with accession number 65221 (http://ds.biogps.org/?dataset=GSE10246&gene=65221).

### Cell isolation and stimulation

Mouse bone marrow cells were collected from femurs and tibias by flushing with Dulbecco’s modified Eagle medium (DMEM; Gibco) containing 10% FBS through a 25-G needle and cultured in DMEM supplemented with 10% FBS, 1% penicillin/streptomycin, and 10 ng/mL rmM-CSF (PeproTech, Rocky Hill, NJ, USA) for 5 days to generate BMDMs. PMs were obtained by intraperitoneal injection of 2 mL sterile 3% thioglycolate broth (Millipore, Billerica, MA, USA). 3 days after injection, cells were harvested by peritoneal lavage with 5 mL cold PBS. The cells were centrifuged and seeded in 12-well plates using DMEM containing 10% FBS and 1% penicillin/streptomycin. After 1 h at 37 °C, nonadherent cells were removed by gently washing three times with warm PBS, and adherent macrophages were used for subsequent experiments^37–39^. BMDMs and PMs were seeded in 12-well plates at a density of 7 × 10^5^ per well, for TLRs activation, the cells were stimulated with 100 ng/mL LPS (*Escherichia coli* O111:B4; Sigma-Aldrich), 1 μg/mL LTA (Lipoteichoic acid from *Bacillus subtilis*; Sigma-Aldrich), 5 μg/mL R837 (Imiquimod, Monmouth Junction, NJ), 1 μM CpG A and 0.2 μM CpG B (Sangon, Shanghai, China) for indicated time. For inhibition studies, the cells were pretreated with 10 μM BAY 11–7082, 50 μM U0126, 10 μM SB203580, 10 μM SP600125 (Beyotime Biotechnology, Shanghai, China) or 2 μM MRT67307 (Monmouth Junction, NJ) for 1 h and then incubated with TLR ligands for another 4 h. Cells were analyzed by real-time PCR and western blot.

### Quantitative real-time PCR

Total RNAs were extracted from cells using the AxyPrep Multisource total RNA Miniprep Kit (Axygen, USA) and the cDNAs generated with PrimeScript^TM^ RT reagent kit (Takara, Japan). The amplification reaction used SYBR *Premix Ex Taq* II (Takara, Japan) followed by detection using qRT-PCR (Applied Biosystems StepOnePlus^TM^). Primers for qRT-PCR were listed in Supplementary Table S1. mRNA expression of target genes was normalized to the housekeeping gene GAPDH, using the ΔCt method and described as relative to GAPDH, ΔCt = average Ct (target gene) - average Ct (GAPDH). To calculate x-fold changes in the expression of target genes, the data were expressed as relative expression using the 2^-ΔΔCt^ method, where ΔΔCt = average ΔCt (treated group) − average ΔCt (untreated or control group).

### Western blotting

Protein samples were collected from macrophages, lysed with Nonidet P40 lysis buffer (50 mM Tris-HCl, 0.4 M NaCl, 0.5% NP-40, 10% glycerol, 1 mM EDTA, pH 8.0), and then centrifuged at 13,000 × *g* × 15 min at 4 °C. Denatured samples were separated by 10% SDS-PAGE (Bio-Rad, USA) and transferred onto nitrocellulose membranes (Millipore, USA). The membranes were blocked at room temperature for 3 h with 5% non-fat milk in Tris-buffered saline with 0.1% Tween 20 (TBST), washed, and then incubated with the primary antibodies: anti-PHT2 antibody (1:500), anti-p38 antibody (1:1000), anti-p-p38 antibody (1:1000), anti-p65 antibody (1:1000), anti-p-p65 antibody (1:1000), anti-Histone H3 antibody (1:1000) and anti-GAPDH antibody (1:10000) at 4 °C overnight. The membranes were washed three times and then incubated with the secondary antibody: goat anti-rabbit or goat anti-mouse IgG-horseradish peroxidase (1:3000; Multi Sciences, China) for 2 h at room temperature. After washing with TBST, three times, the bound antibody was incubated with ECL Western Blotting Substrate (GE Healthcare, USA) and detected by the Alpha FluorChem E System (ProteinSimple, USA). Protein amount was quantitated by densitometry using Image J (verision 1.4.3.67, USA) and protein abundance was expressed relative to GAPDH. Antibodies used were against mouse SLC15A3 from a co-author (Dr. David E. Smith, University of Michigan, USA), GAPDH from MultiSciences (Hangzhou, China) and p-p38, p38, p-p65, p65, Histone H3 from Cell Signal Technology (Beverly, MA, USA). The polyclonal antibody against human SLC15A3 was generated by HUABIO (Hangzhou, China). Blots were subjected to scanning densitometry, and protein abundance was expressed relative to that of the maximal observed expression

### Plasmid construction

The 5′ upstream region (2.5 kb) of the mouse Slc15a3 gene was amplified from genomic DNA isolated from BMDMs using the forward and reverse primers for SLC15A3-Pro (Supplementary Table S1). Truncated segments of mouse Slc15a3 promotor (P1, P2, P3) were amplified with specific primers (Supplementary Table S1), digested by Sac I and Sma I restriction enzymes (ThermoFisher, USA), and then cloned into pGL3-basic vector (Promega, Germany). Mouse NF-κB plasmid was constructed with specific primers using BMDM cDNA as a template (Supplementary Table S1). PCR products were digested by EcoR I and Xba I (ThermoFisher, USA), and then cloned to pcDNA3.1( + ) plasmid (Invitrogen, USA).

### Reporter gene assay

HeLa cells were seeded in 24-well plates 1 day prior to transfection using Lipofectamine 2000 (Invitrogen, USA) according to the manufacturer’s instructions. Cells were transfected with 0.25 μg of either pGL3-basic vector or pGL3-basic-SLC15A3 promotor reporter constructs. In all cases, 0.025 μg of pRL-TK plasmid (Promega, Germany) was cotransfected for normalization of transfection efficiency. Overexpression of NF-κB was accomplished by co-transfection with 0.25 μg pcDNA3.1( + )-mNF-κB plasmid. Cells were harvested 24 h after transfection, and luciferase activity was measured by a Reporter Assay System Kit (Promega, Germany) and then expressed as x-fold activation relative to the activity of non-mNF-κB transfected cells.

### ELISA for cytokines

Cell culture supernatants and serum were collected, and the levels of IL-6 and TNF-α were determined using ELISA kits (R&D Systems, USA) according to the manufacturer’s instructions.

### siRNA transfection

Sequences of siRNA specifically targeting the SLC15A3 gene were designed through Sigma-Aldrich (shRNA ID TRCN0000044619 and shRNA ID TRCN0000044620), and synthesized by GenePharma (GenePharma Co., Ltd, Shanghai, China). The sequences were as follows: 5′-CUUUGGGAACAUCAACAAU-3′ for #1 and 5′-GGAUGGUCUACUUCCAGAUTT-3′ for #2. THP-1 cells were cultured and differentiated into macrophages in RPMI-1640 medium containing 100 ng/mL PMA for 48 h. The cells were then transfected with control nontargeting siRNA or specific SLC15A3 siRNA using jetPrime transfection reagent (Polyplus transfection, NY, USA) according to the manufacturer’s instructions. After transfecting for 24 h, the cells were treated with 100 ng/mL LPS for specified time periods, and the protein levels of IL-6 and TNF-α were determined by ELISA or protein levels of p38, p-p38, p65 and p-p65 were determined by Western blotting.

### *E. coli*-induced peritonitis

To induce peritonitis, mice were injected intraperitoneally with 1 × 10^7^ cfu/mL *E. coli*-56809 (0.5 mL/25 g body weight) dissolved in sterile PBS. After being challenged by *E. coli* for specified time periods, the mice were sacrificed by ether inhalation and the serum harvested for analysis of IL-6 and TNF-α protein expression by ELISA. Peritoneal macrophages were collected by washing peritoneal cavities with cold PBS. The mRNA and protein expression of SLC15A3, IL-6 and TNF-α were determined by real-time PCR and Western blotting, respectively.

### Statistical analysis

Data are expressed as mean ± SE of three independent experiments with each experiment being carried out at least in triplicate. Statistical comparisons between two groups were determined by Student’s unpaired two-tailed *t* test, and among multiple treatment groups were determined by one-way ANOVA followed by either Tukey’s or Dunnett’s test (GraphPad Prism, v6.0; GraphPad Software, Inc., La Jolla, CA). A probability of *P* ≤ 0.05 was considered to be statistically significant.

## Electronic supplementary material

Supplementary Information 1

Supplementary Figure S1

Supplementary Figure S2

Supplementary Figure S3

Supplementary Information 2
