# Supplementary material for: Regulation and biological role of the peptide/histidine transporter SLC15A3 in Toll-like receptor-mediated inflammatory responses in macrophage
Source: Cell Death Dis. 2018 Jul 10;9(7):770. doi: 10.1038/s41419-018-0809-1 (PMC6039463; doi:10.1038/s41419-018-0809-1)
Supplement: Supplementary file 1 — Supplementary Information 1 [file 41419_2018_809_MOESM1_ESM.docx]

**Supplementary Table S1 Sequences for primers used in this study**

| Target gene | Sequence |
| --- | --- |
| **q-PCR primers** | |
| mouse-SLC15A3-F | GCTGAAGCTTGCGTTCCAA |
| mouse-SLC15A3-R | AACAGGTGGGCACTTTCAGAGT |
| mouse-IL-6-F | TCCTCTCTGCAAGAGACTTCCATCC |
| mouse-IL-6-R | GGGAAGGCCGTGGTTGTCACC |
| mouse-TNFα-F | CCACGTCGTAGCAAACCACCAAG |
| mouse-TNFα-R | ATCCATGCCGTTGGCCAGGAG |
| mouse-IFNβ-F | CGGACTTCAAGATCCCTATGGA |
| mouse-IFNβ-R | TGGCAAAGGCAGTGTAACTCTTC |
| mouse-GAPDH-F | GAGACAGCCGCATCTTCTTGT |
| mouse-GAPDH-R | CACACCGACCTTCACCATTTT |
| human-SLC15A3-F | AGGGCAGCAGCTACACGGAGT |
| human-SLC15A3-R | GGACAGTGGTGCCGCGTTGT |
| human-IL-6-F | GGTACATCCTCGACGGCATCT |
| human-IL-6-R | GTGCCTCTTTGCTGCTTTCAC |
| human-TNFα-F | CCCAGGCAGTCAGATCATCTTC |
| human-TNFα-R | AGCTGCCCCTCAGCTTGA |
| human-GAPDH-F | GCACCGTCAAGGCTGAGAAC |
| human-GAPDH-R | TGGTGAAGACGCCAGTGGA |
| **Primers for promoter luciferase reporter** | |
| SLC15A3-P1-F | AATGAGCTCAGAGGACCAGGCACTCATGT |
| SLC15A3-P1-R | AATCCCGGGTTCCCAGTGCTTAGCAAT |
| SLC15A3-P2-F | AGAGAGCTCGTGTTTGATGGTGACTCTGGC |
| SLC15A3-P2-R | AATCCCGGGACACTGAGAGGCATCTTCT |
| SLC15A3-P3-F | AATGAGCTCTGGACTGGTCTCAGCGTCA |
| SLC15A3-P3-R | AATCCCGGGGTGTCGTTCTCTCTCTCTT |
| SLC15A3-Pro-F | CTCGAGCTCTCAGCTCTCATTCCACTCATTCG |
| SLC15A3-Pro-R | AATCCCGGGTGGCCGTCCCAATTGAAGT |
| NF-κB-F | AATGAATTCTGTAACTGGAGTTTGACGGTCG |
| NF-κB-R | CGCTCTAGACTAAATTTTGCCTTCAATAGGTC |
