# Supplementary figures and images for: Regulation and biological role of the peptide/histidine transporter SLC15A3 in Toll-like receptor-mediated inflammatory responses in macrophage
Source: Cell Death Dis. 2018 Jul 10;9(7):770. doi: 10.1038/s41419-018-0809-1 (PMC6039463; doi:10.1038/s41419-018-0809-1)

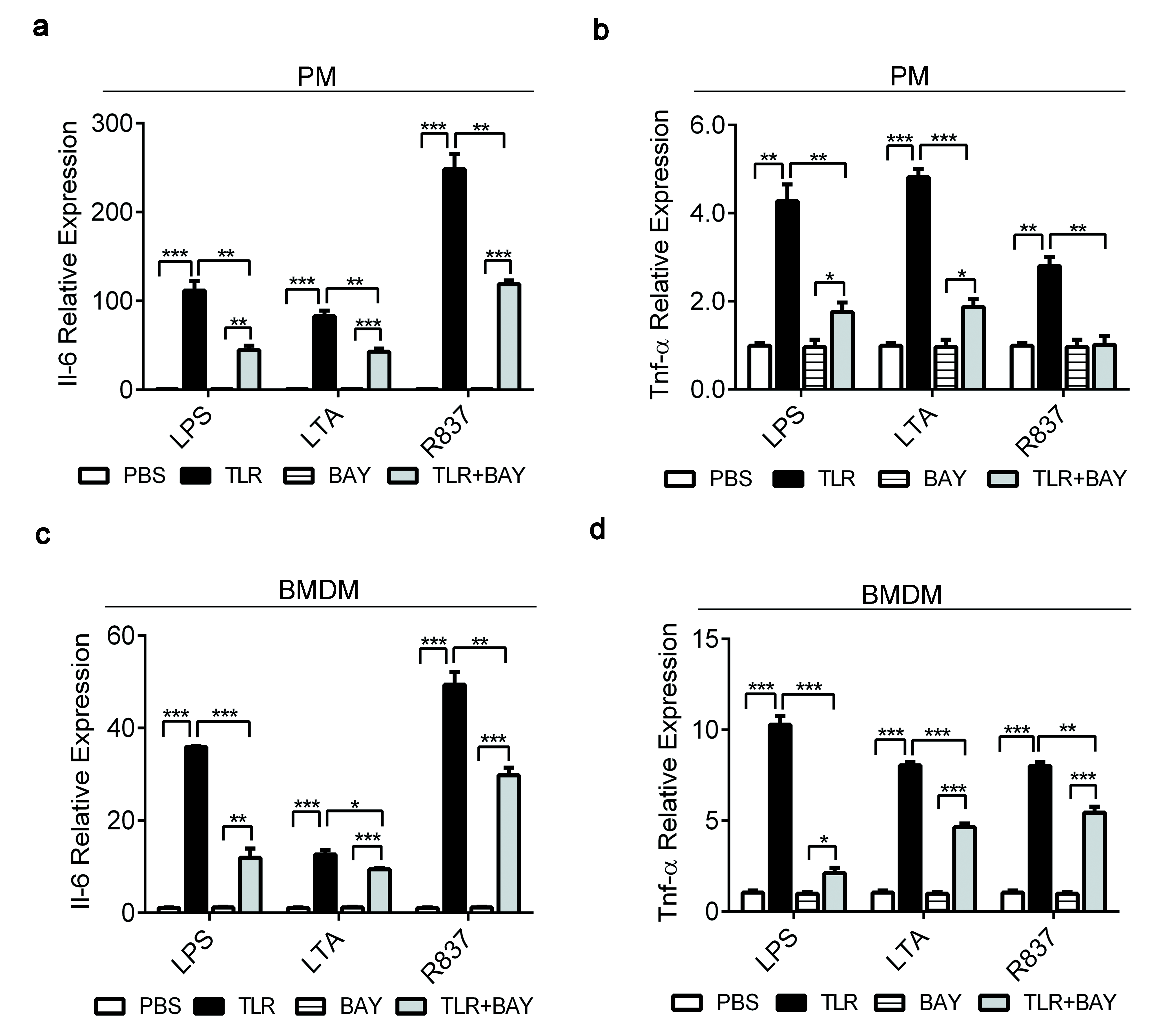

Supplement: Supplementary file 2 — Supplementary Figure S1 [file 41419_2018_809_MOESM2_ESM.tif]

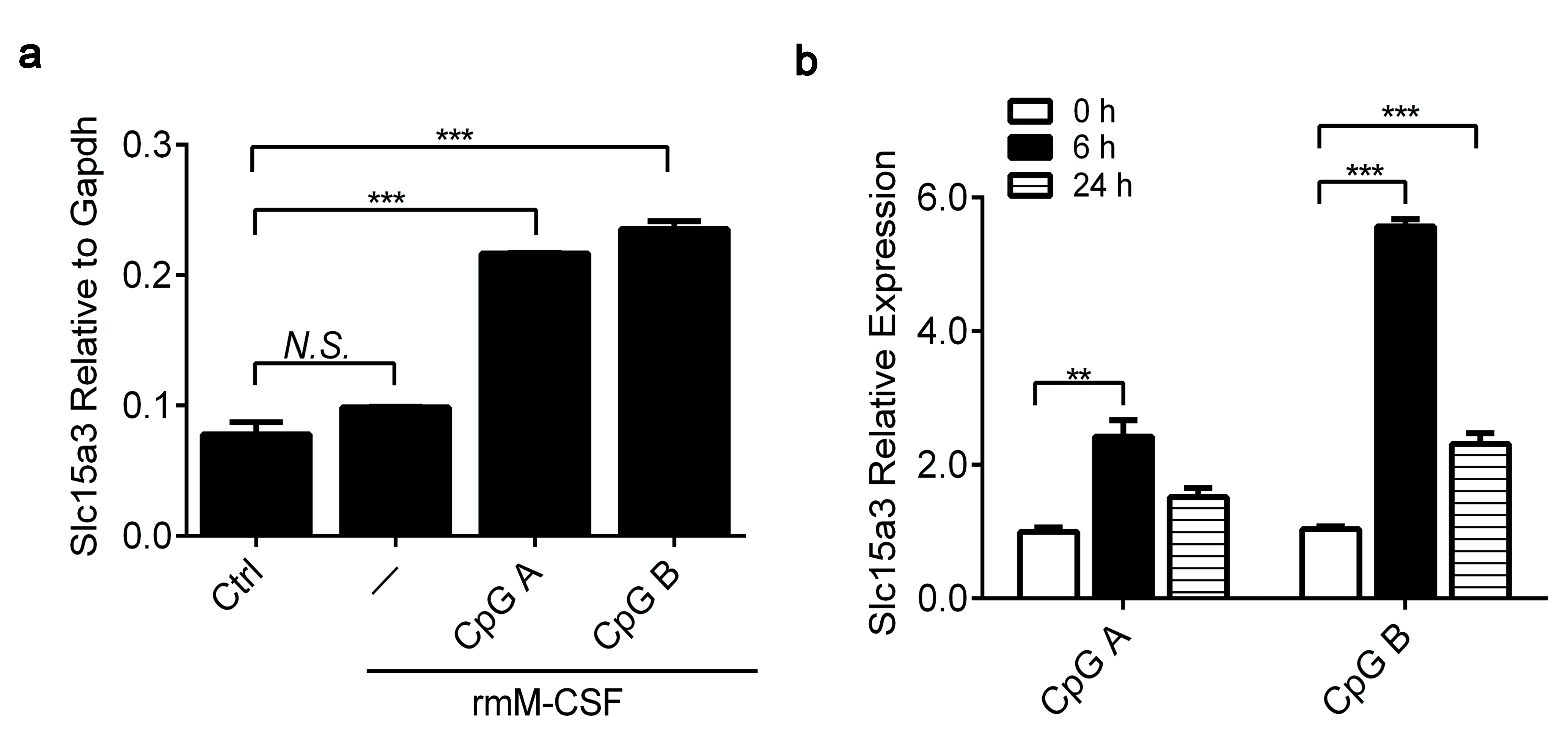

Supplement: Supplementary file 3 — Supplementary Figure S2 [file 41419_2018_809_MOESM3_ESM.tif]

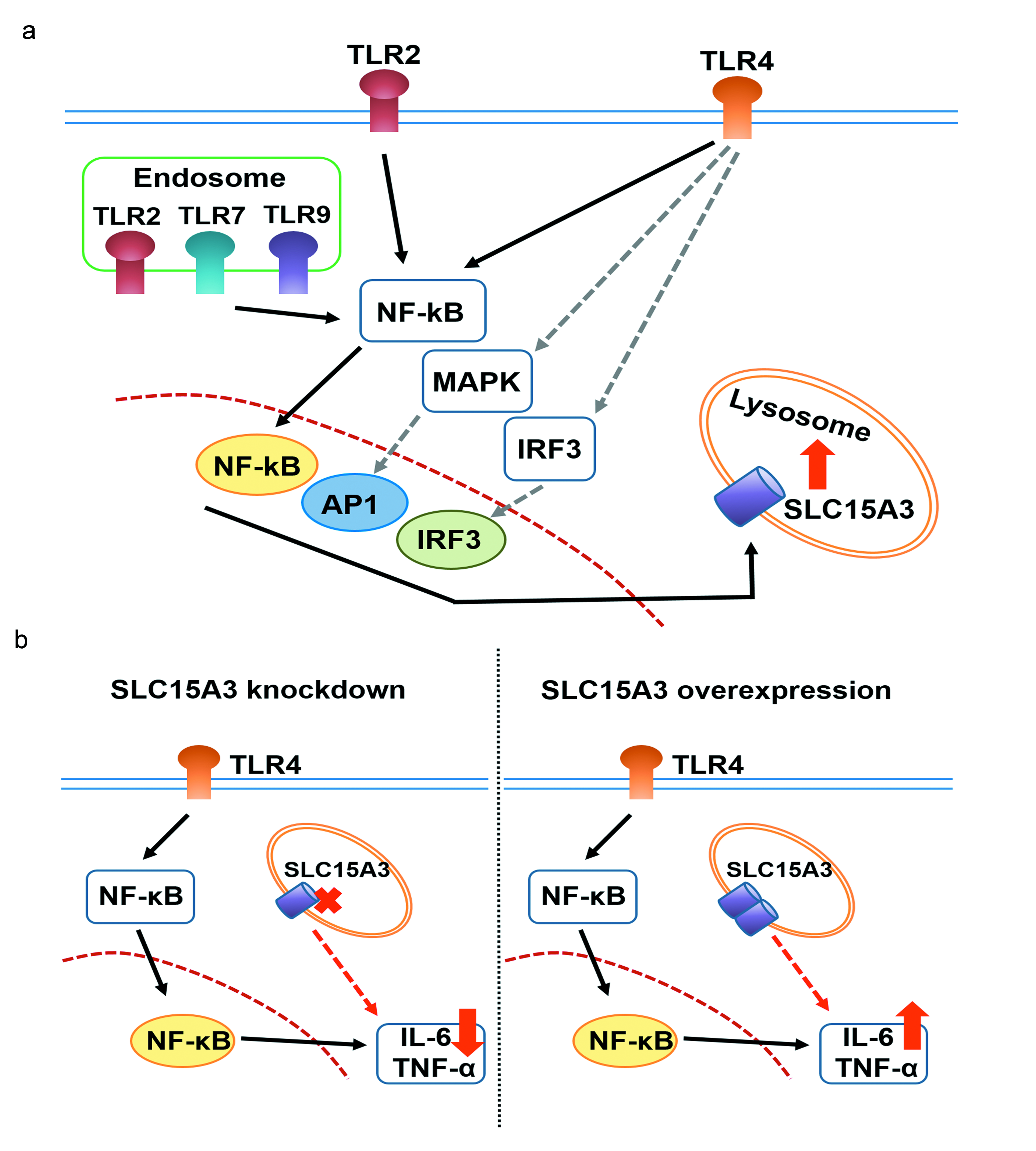

Supplement: Supplementary file 4 — Supplementary Figure S3 [file 41419_2018_809_MOESM4_ESM.tif]
