# Supplementary material for: Regulation and biological role of the peptide/histidine transporter SLC15A3 in Toll-like receptor-mediated inflammatory responses in macrophage
Source: Cell Death Dis. 2018 Jul 10;9(7):770. doi: 10.1038/s41419-018-0809-1 (PMC6039463; doi:10.1038/s41419-018-0809-1)
Supplement: Supplementary file 5 — Supplementary Information 2 [file 41419_2018_809_MOESM5_ESM.docx]

**Supplementary Figure Legends**

**Supplementary Figure S1**

TLR ligands induced the upregulation of proinflammatory cytokines in mouse PMs and BMDMs. mRNA expression of Il-6 and Tnf-α in mouse PMs (**a**, **b**) and BMDMs (**c**, **d**) pretreated with or without 10 μM BAY 11-7082 (BAY) for 1 h, and then treated with or without 100 ng/mL LPS, 1 μg/mL LTA, or 5 μg/mL R837 for another 3 h. One-way ANOVA followed by Tukey’s test was used to evaluate the statistical differences, ^*^*P* < 0.05, ^**^*P* < 0.01, and ^***^*P* < 0.001. Data are expressed as mean ± SE (n=3).

**Supplementary Figure S2**

CpG A and CpG B induces the upregulation of Slc15a3 expression in BMDMs. **a** mRNA expression of Slc15a3 in BMDMs stimulated with 1 μM CpG A or 0.2 μM CpG B in the presence of rmM-CSF for 6 h. **b** mRNA expression of Slc15a3 in mouse BMDMs treated with 1 μM CpG A or 0.2 μM CpG B for specified periods of time. One-way ANOVA followed by Dunnett’s test was used to evaluate the statistical differences.^*^*P* < 0.05, ^**^*P* < 0.01, and ^***^*P* < 0.001 compared with 0 h. Data are expressed as mean ± SE (n=3).

**Supplementary Figure S3**

Regulation and role of SLC15A3 in TLR signaling. **a** SLC15A3 can be regulated by various TLR ligands. SLC15A3 was upregulated by TLR2, TLR4, TLR7 and TLR9 ligands in macrophages at both the mRNA and protein levels. The upregulation of SLC15A3 by TLR ligands could be attenuated by the inhibitors of NF-κB, MAPK and TRIF. **b** Knockdown or overexpression of SLC15A3 has an effect on the TLR4-triggered immune responses. Knockdown of SLC15A3 in THP-1 cells significantly reduced LPS-induced upregulation of IL-6 and TNF-α. In turn, overexpression of SLC15A3 in A549 cells significantly enhanced LPS-triggered IL-6 production.

**
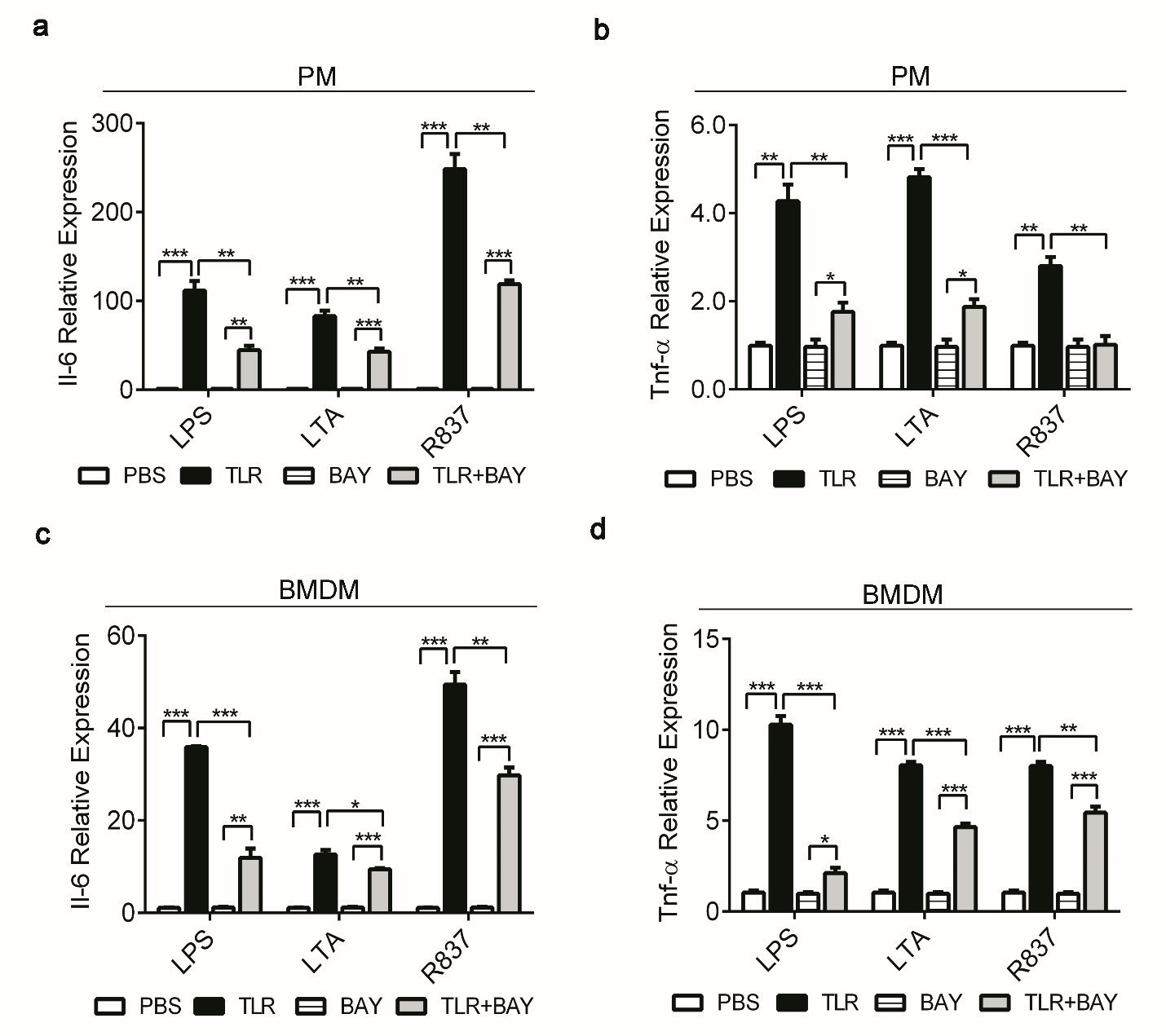
**

**Supplementary Figure S1**

**
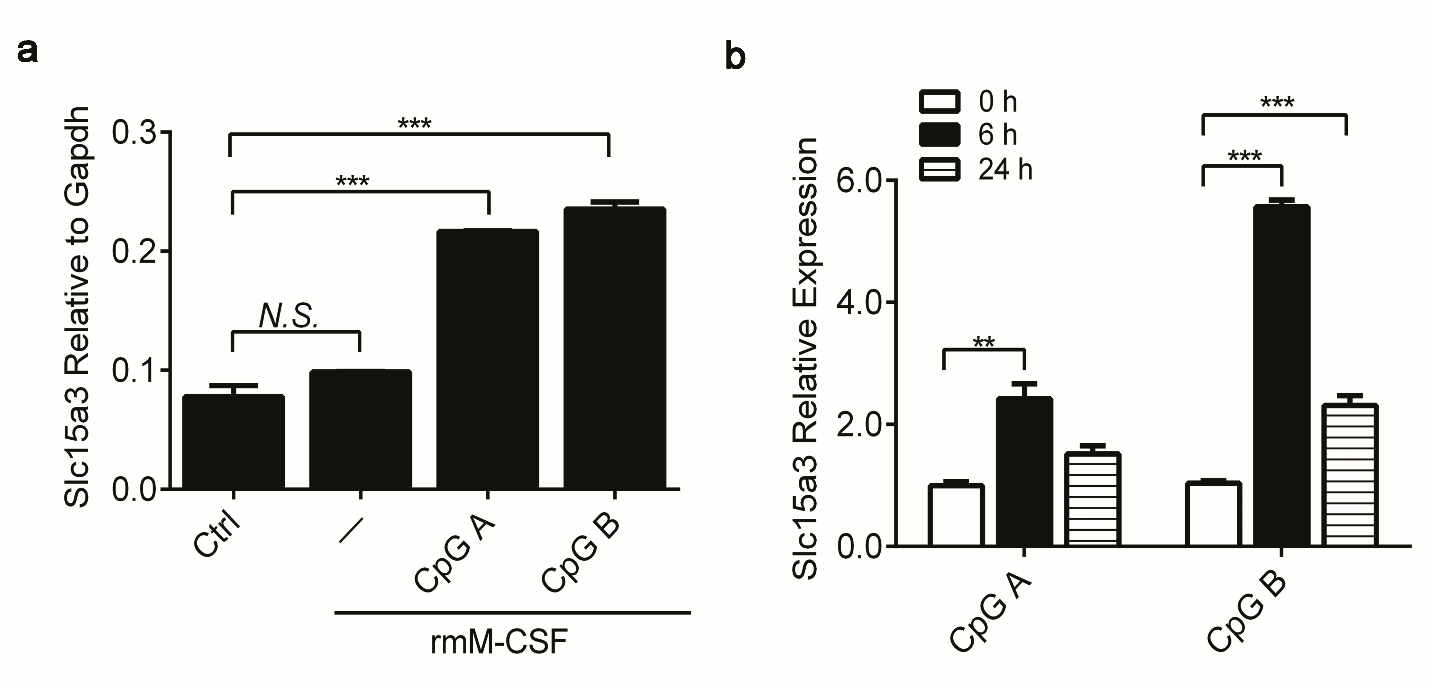
**

**Supplementary Figure S2**

**
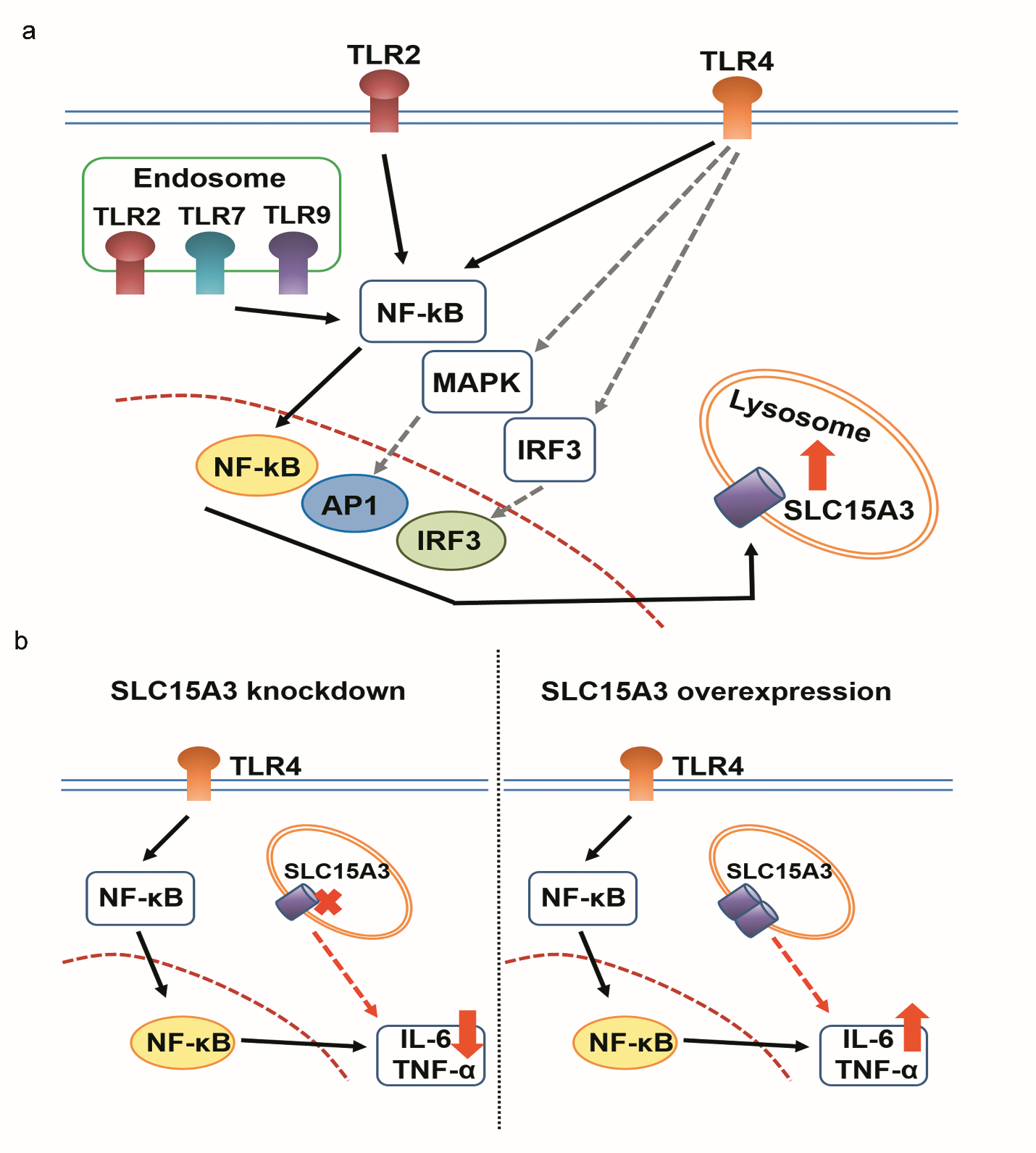
**

**Supplementary Figure S3**
